# MiR-146a expression profiles in osteoarthritis in different tissue sources: a meta-analysis of observational studies

**DOI:** 10.1186/s13018-022-02989-7

**Published:** 2022-03-05

**Authors:** Jia-Neng Liu, Song Lu, Chang-Ma Fu

**Affiliations:** Department of Orthopedics, The Third People’s Hospital of Hefei, No. 204, Wangjiang East Road, Baohe District, Hefei, 230041 Anhui China

**Keywords:** Osteoarthritis, MIR146A, MicroRNA, Diagnosis, Meta-analysis

## Abstract

**Background:**

MiR-146a has been widely studied in the pathogenesis of osteoarthritis (OA); however, the results are still controversial.

**Objective:**

This meta-analysis analyzes the expression profile of miR-146a in various tissues of OA patients.

**Methods:**

Public databases were searched for appropriate studies published up to September 1, 2021. A case–control study comparing the OA population and a non-OA healthy population was included.

**Results:**

26 articles were included in analysis. The results showed that the expression level of miR-146a in peripheral blood mononuclear cells (PBMCs) was significantly higher in OA patients than in controls (SMD: 1.23; 95% CI 0.08–2.37; *p* = 0.035) but not in plasma (SMD: 1.09; 95% CI − 0.06, 2.24; *p* = 0.064). The expression level of miR-146a in cartilage was also significantly higher in OA patients than in controls (SMD: 6.39; 95% CI 0.36, 12.4; *p* = 0.038) but not in chondrocytes (SMD: − 0.71; 95% CI − 4.15, 2.73; *p* = 0.687). The miR-146a level was significantly lower in synoviocytes in the OA population than in control patients (SMD: − 0.97; 95% CI − 1.68, − 0.26; *p* = 0.008). In synovial tissue, synovial fluid, and regulatory T cells, there was no significant difference.

**Conclusion:**

The expression level of miR-146a in cartilage tissue and PBMCs was significantly higher in OA patients than in non-OA healthy controls. Due to the limitations of this study, more research is needed to confirm these results in the future.

*Trial registration*: retrospectively registered.

**Supplementary Information:**

The online version contains supplementary material available at 10.1186/s13018-022-02989-7.

## Background

Osteoarthritis (OA) is a type of degenerative joint disease with a high incidence in the elderly population and is also the most common cause of activity restriction in adults. OA affects 7% of the world's population, while it is estimated that more than 240 million people suffer from symptomatic activity-restricted OA [1]. Population aging and an increasing proportion of obese people may lead to a further increase in OA morbidity [2]. The diagnosis of OA mostly depends on clinical symptoms, and there is still a lack of specific biomarkers at present.

MicroRNAs are noncoding RNAs that are highly conserved and tissue specific and can inhibit gene translation into proteins and promote mRNA degradation to achieve posttranscriptional regulation. The complex interactions between microRNAs and their target genes play an important role in the pathogenesis and development of OA [3].Of them, miR-146a has been widely studied for the pathogenesis of OA. Basic research suggests that miR-146a promotes chondrocyte apoptosis, cartilage damage, inflammation, and neovascularization [4, 5]. It also suggested that miR-146a has the potential to be a biomarker for predicting and diagnosing OA [6]. However, for OA, the expression profile of miRNAs may be different in various tissues.

Recently, a study considered that the level of circulating miR-146a is upregulated in patients with OA [7]. For the cartilage and bone tissues of OA patients, the expression of miR-146a was significantly downregulated [8]. The exosomes of synovial fluid from OA patients also contain miR-146a, which is highly expressed in the early stage and decreases in the later stage of disease [9, 10]. At present, although many studies have shown that miR-146a is significantly related to the pathogenesis of OA, its expression level is controversial [11]. Furthermore, its expression profile in various tissues and its diagnostic value for OA still need to be evaluated in detail. This meta-analysis was performed to analyze the expression profile of miR-146a in various tissues of OA patients and evaluate its potential application value as a biomarker.

## Methods

### Search strategy

Two researchers independently searched the PubMed, Embase, Cochrane, Scopes, Reaxys, and Ebscohost databases. These databases were used to obtain all the appropriate studies published up to September 1, 2021. The key words included miR-146a-related items (miR-146a, MIR146, miR-146a-3p, miR-146a-5p, MIR146A, MIRN146, MIRN146A, miRNA146A) and OA-related items (osteoarthritis, osteoarthritis, osteoarthrosis). The retrieval strategy adopts the union of the above two types of items. Retrieval does not set language restrictions. Manual retrieval was performed for the references of important reviews to prevent omission.

### Inclusion and exclusion criteria

Two researchers independently screened the items adopted from the search results. The inclusion criteria were as follows: 1. case–control study; 2. study comparing the OA population and nonOA healthy population; and 3. study that obtained miR-146a expression profiles, miR-146a-related single nucleotide polymorphisms (SNPs) between two groups, or report diagnostic accuracy of OA with miR-146a. The exclusion criteria were as follows: 1. repeated reports; 2. studies on the changes in miR-146a expression before and after OA treatment; 3. studies that did not report miR-146a expression results of a nonOA healthy population; 4. studies comparing OA and rheumatoid arthritis (RA) populations; 5. studies that did not report specific miR-146a expression levels, SNP carrier frequency, or diagnostic accuracy results; and 6, basic research based on animal or cellular levels. Case reports, editorials, and expert opinions were also excluded.

### Data extraction and quality assessment

Two authors independently extracted data from each eligible study using a predefined data collection sheet, which included the first author’s name, publication year, study location, sample size, and test tissue source. Any of following outcomes was recorded: fold-change of miR-146a expression between OA and nonOA health groups by quantitative polymerase chain reaction (qPCR); SNP-related study reported the carrier frequencies of OA and control population in each gene phenotype; the sensitivity, specificity and 2 × 2 table reported in the diagnostic accuracy study. The results were extracted from figures if specific data were not reported. The QUADAS-2 tool is used to evaluate the quality of eligible studies and consists of four domains: patient selection, index test, reference standard, and flow and timing [12].

### Statistical analysis

The expression profiles, SNP results, and diagnostic accuracy results will be analyzed. The expression profiles are also reported according to different tissue sources. The continuous data were pooled by standardized mean difference (SMD) with its 95% confidence interval (CI). The binary outcomes were pooled by odds ratio (OR) with its 95% (CI).The Paule-Mandel estimator is used to estimate the between-study variance τ^2 [13]. With respect to heterogeneity, the *I*^2^ statistic was used to estimate the degree of heterogeneity among the studies. When *I*^2^ ≥ 50%, a random effects model was adopted; otherwise, a fixed effects model was adopted. Publication bias was also evaluated by using funnel plots, Egger’s test, and Begg’s test. If there was publication bias, the trim and fill method was used to complement the potential study to make the funnel plot symmetrical and to analyze the impact on the pooling results.

We analyzed the diagnostic power of microRNAs in the diagnosis of OA by pooling sensitivity and specificity. Summary receiver operating characteristic (SROC) curves and the area under the curve (AUC) were calculated if feasible [14]. For SNP-related outcomes, the association analyses were performed using the following five genetic models: allelic (W vs. M), dominant (WW + WM vs. MM), recessive (WW vs. WM + MM), heterozygous (WM vs. MM), and homozygous (WW vs. MM). W represents the major wild-type allele, and M represents the minor mutant-type allele [15]. The odds ratios with 95% CIs were calculated to assess the associations. R program (version 4.1.0) and RevMan 5.3 were used to perform the analysis.

## Results

**Fig. 1:**
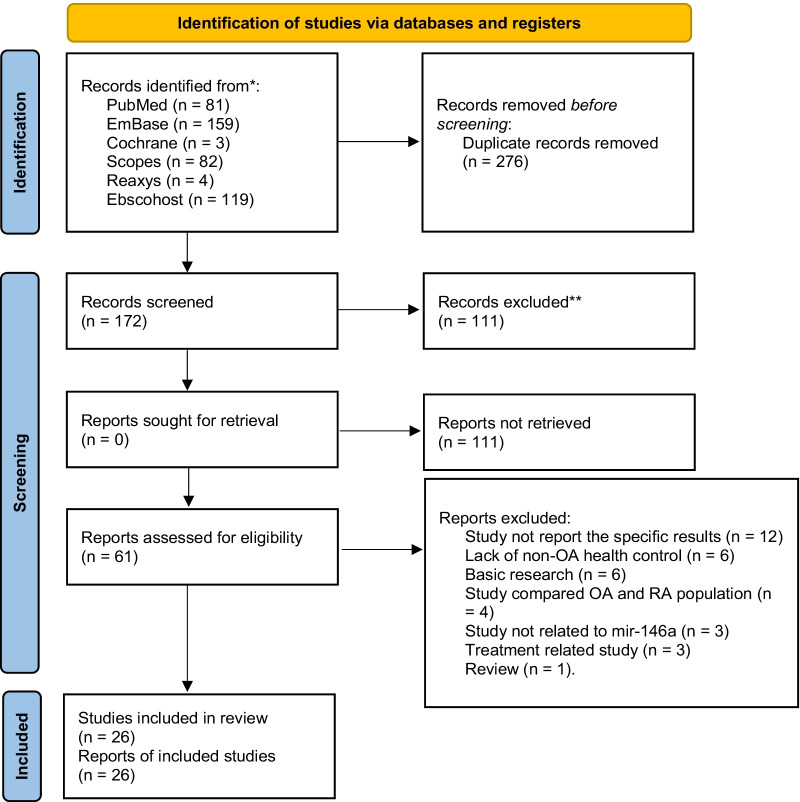
PRISMA flowchart of the literature search and study selection process

A total of 172 items were identified through databases after removing duplications. After screening the titles and abstracts, 111 items were excluded, and 61 items were obtained to view the full-text article. The following studies were excluded due to: study not report the specific results or results could not be extracted (12); lack of nonOA health control (6); basic research (6); study compare OA and RA population (4); study not related to mir-146a (3); study only report the changes of miR-146a expression before and after OA treatment (3); review (1). Finally, 26 articles were included in the analysis [16–41]. The selection process of eligible study was detailed in the Preferred Reporting Items for Systematic Reviews and Meta-Analysis (PRISMA) flow diagram (Fig. 1). The review was reported according with PRISMA guidelines (Additional file 1: PRISMA Checklist).

The included studies were published from 2010 to 2021 and included two large-scale SNP-related studies [16, 24] and two reported diagnostic accuracy results [18, 27]. The tissue source for the miR-146a test included plasma, regulatory T cells, peripheral blood mononuclear cells (PBMCs), local synovial fluid, synovial tissue, synoviocytes, cartilage tissue, and chondrocytes. It should be noted that the people who obtain synovial tissue/cells and cartilage tissue/cells are often those who have experienced joint replacement surgery because of severe OA. Additionally, synoviocytes and chondrocytes have undergone extraction and even culture in vitro. Therefore, these types of cells are analyzed separately from synovial and cartilage tissue, which are only through the process of tissue separation (Table 1).

**Table 1:** Characteristic of included studies

Study	Study location	Sample size	Tissue source
Miranda-Duarte et al. [16]	Mexican mestizos	689	PBMCs (SNP-related study)
Nie et al. [17]	China	132	Synovial fluid
Wu et al. [18]	China	70	Plasma
Zhang et al. [19]	China	44	Cartilage
Kmiolek et al. [20]	Poland	26	Treg cells
Rousseau et al. [21]	France	133	Plasma
Shao et al. [22]	China	40	Cartilage
Wang et al. [23]	China	610	Plasma
Papathanasiou et al. [24]	Greece	1688	PBMCs (SNP-related study)
Papathanasiou et al. [25]	Greece	35	Chondrocytes/synoviocytes
Skrzypa et al. [26]	Poland	30	Cartilage/serum
Ali et al. [27]	India	28	Serum
Budd et al. [28]	UK	22	Chondrocytes
Cheleschi et al. [29]	Italy	10	Chondrocytes
Kopanska et al. [30]	Poland	31	Cartilage
Soyocak et al. [31]	Turkey	150	PBMC
Mu et al. [32]	China	60	Synovial fluid
Zakaria et al. [33]	Egypt	56	PBMC
Xu et al. [34]	China	20	Synovial fluid
Wang et al. [35]	China	14	PBMC
Qian et al. [36]	China	30	PBMC
Abou-Zeid et al. [37]	Egypt	105	PBMC
Okuhara et al. [38]	Japan	72	PBMC
Murata et al. [39]	Japan	68	Plasma
Niimoto et al. [40]	Japan	11	PBMC
Nakasa et al. [41]	Japan	6	Synovial tissue

*PBMCs* peripheral blood mononuclear cells, *SNP* single nucleotide polymorphism, *Treg cells* regulator T cells

**Fig. 2:**
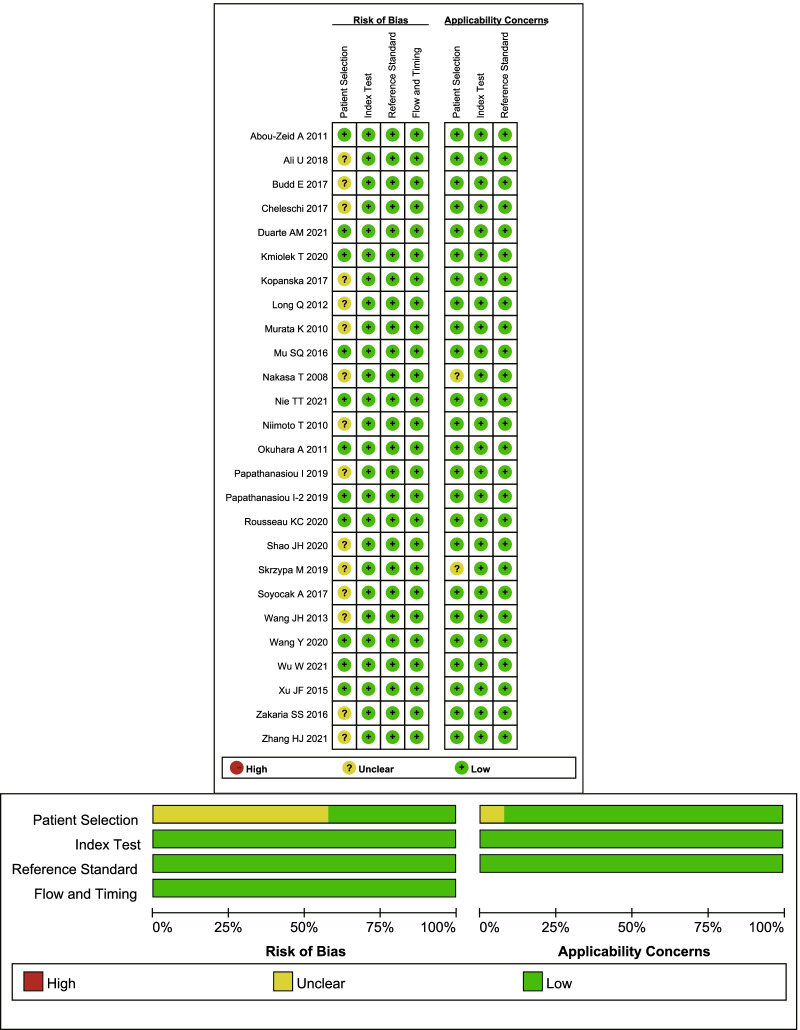
Risk of bias graph for included primary studies

For the evaluation of the overall quality, the following factors will affect the risk of bias. Whether the patients were included consecutively or randomly was not described in detail in most studies. Because this meta-analysis is mostly based on case–control studies, the items that “avoid case–control design” are not applicable. In addition, another potential bias was that if the study used joint tissue as the sample source, only people who tended to undergo joint replacement surgery for severe OA were included. However, this study used blood and synovial fluid as sample sources that tended to include a wider range of OA populations. The overall quality of the included studies is shown in Fig. 2.

**Fig. 3:**
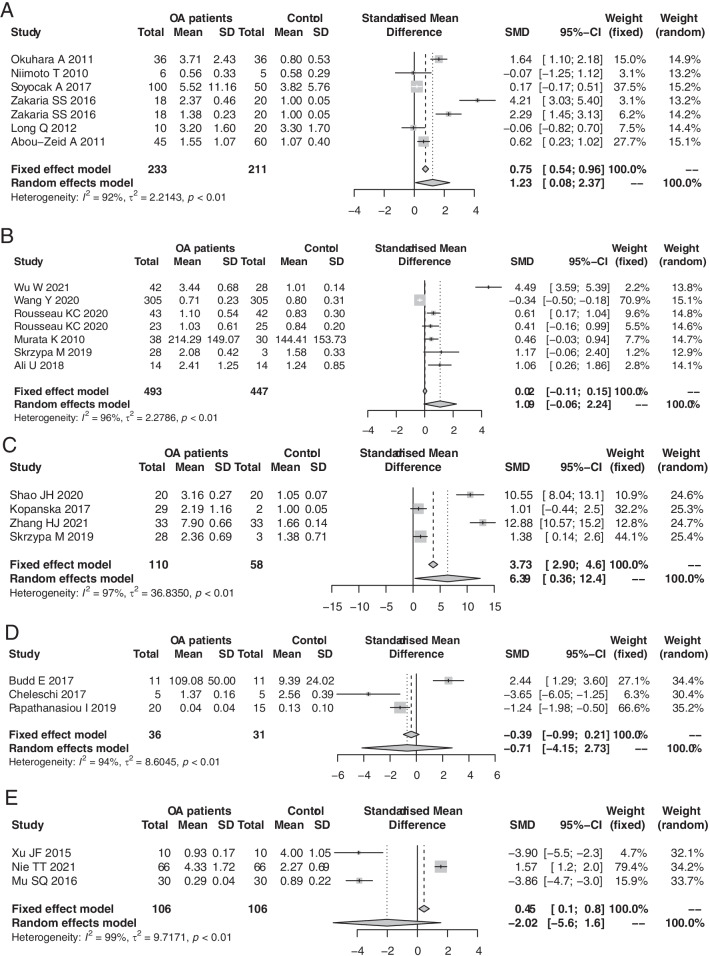
Forest plots for the comparisons of miR-146a expression between OA patients and no-OA health control in PBMCs (**A**), plasma (**B**), cartilage (**C**), chondrocytes (**D**), and synovial fluid (**E**)

**Fig. 4:**
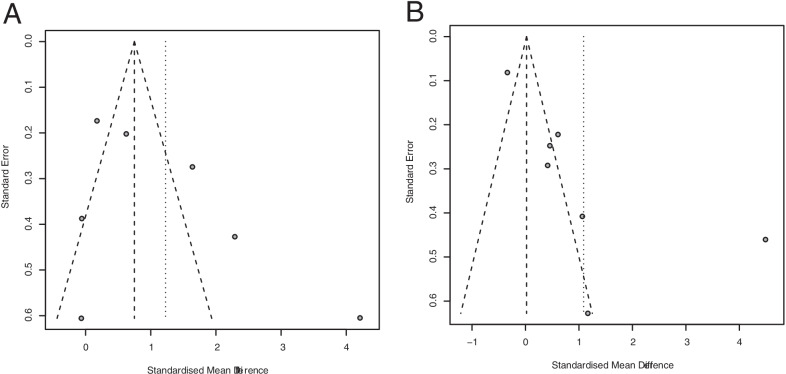
Funnel plots for the comparisons of miR-146a expression in PBMCs (**A**) and plasma (**B**)

Seven studies used PBMCs as sample source. A random effects model showed that the expression level of miR-146a in PBMCs was significantly higher in OA patients than in controls (SMD: 1.23; 95% CI 0.08–2.37; *p* = 0.035) (Fig. 3A). The results did not detect potential publication bias (Begg’s test, *p* = 0.177; Egger’s test, *p* = 0.206) (Fig. 4A).

Seven studies used plasma or serum as a sample source. A random effects model showed that the miR-146a expression level was not significantly different between the OA and control groups (SMD: 1.09; 95% CI − 0.06, 2.24; *p* = 0.064) (Fig. 3B). Then, publication bias existed (Begg’s test, *p* = 0.453; Egger’s test, *p* = 0.024). After trim and fill method modification, no significant difference was detected (SMD: − 0.11; 95% CI − 1.47, 1.25; *p* = 0.877) (Fig. 4B).

Four studies used cartilage tissue as a sample source. The pooling results based on a random effects model showed that the level of miR-146a in OA cartilage was significantly higher than that in nonOA cartilage (SMD: 6.39; 95% CI 0.36, 12.4; *p* = 0.038) (Fig. 3C). Three studies used chondrocytes as a sample source, and a random effect model showed that there was no significant difference between OA patients and controls (SMD: − 0.71; 95% CI − 4.15, 2.73; *p* = 0.687) (Fig. 3D). Whether the chondrocyte isolation process impacts the miR-146a level needs further research confirmation.

Three studies researched synovial fluid, but with opposite results (I^2^ = 99%, *p* < 0.01). The pooling results showed that there was no significant difference in miR-146a levels between the OA and control groups (SMD: − 2.02; 95% CI − 5.60, 1.60; *p* = 0.27) (Fig. 3E). Other results were based on a single study. These results showed that the miR-146a level was significantly lower in synoviocytes from the OA population than in synoviocytes from the control population (SMD: − 0.97; 95% CI − 1.68, − 0.26; *p* = 0.008). In synovial tissue and regulatory T cells, there was no significant difference.

In the PBMC results, subgroup analysis was performed in Kellgren-Lawrence (KL) grade 2–3 and KL grade 3–4 populations. A random effects model showed that miR-146a expression was significantly higher in OA patients than in controls in both the KL grade 2–3 subgroup (SMD: 1.84; 95% CI 0.37, 3.31; *p* = 0.014) and the KL grade 3–4 subgroup (SMD: 1.32; 95% CI 0.31; 2.32; *p* = 0.010). However, there was no statistical significance between the two subgroups (*p* = 0.56).

Two studies analyzed the diagnostic accuracy of plasma miR-146a for OA [18, 27]. The pooling sensitivity and specificity results were 0.80 (95% CI 0.68, 0.89) and 0.93 (95% CI 0.80, 0.98), respectively. It is suggested that plasma miR-146a has certain diagnostic ability for OA. However, based on the above results that the plasma miR-146a level was not significantly different between OA patients and controls, the diagnostic accuracy of plasma miR-146a was suspected.

Two studies focused on SNPs of miR-146a [16, 24]. For the rs2910164 locus, there were no significant results in any model [allele model (OR: 1.09; 95% CI 0.95, 1.24; *p* = 0.205); heterozygous model (OR: 1.14; 95% CI 0.96, 1.35; *p* = 0.125); homozygous model (OR: 1.08; 95% CI 0.75,1.56; *p* = 0.690); dominant model (OR: 1.13; 95% CI 0.96, 1.34; *p* = 1.32); recessive model (OR: 1.03; 95% CI 0.72, 1.47; *p* = 0.879)]. For rs57095329 locus, only one study reported. There were still no significant results [allele model (OR: 1.11; 95% CI 0.78, 1.58; *p* = 0.577); recessive model (OR: 1.12; 95% CI 0.77, 1.63; *p* = 0.554)].

## Discussion

**Fig. 5:**
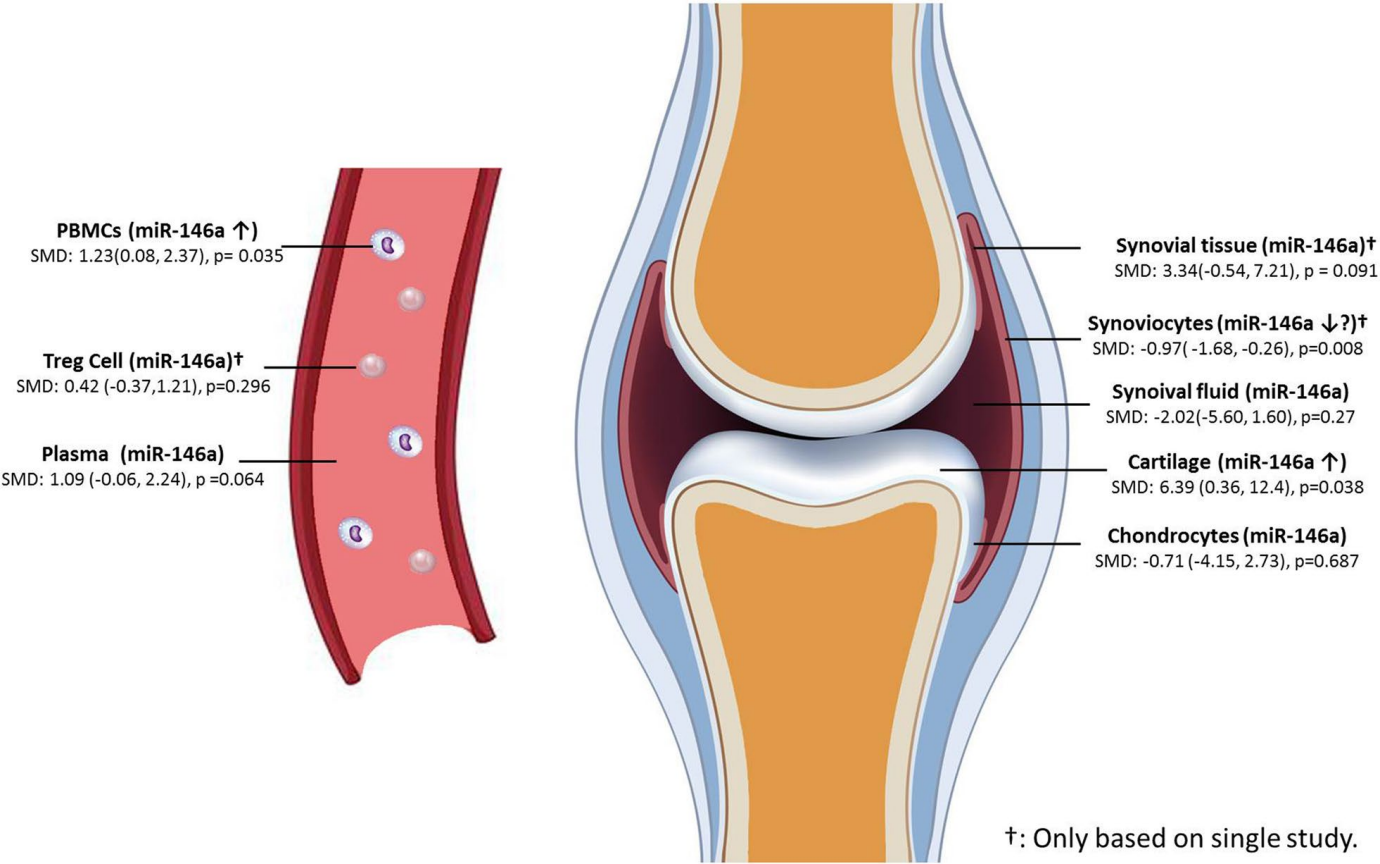
Schematic diagrams illustrating miR-146a expression in different tissue origin of OA patients

This meta-analysis first analyzed the difference in miR-146a expression between OA and nonOA control patients in various tissue samples. The results showed that the expression level of miR-146a in PBMCs was significantly higher in OA patients than in nonOA healthy people. However, there was no significant difference in plasma. The expression level of miR-146a in cartilage tissue was significantly higher in OA patients than in controls. However, no significant difference was found in chondrocytes. Based on a single study, the expression of miR-146a in synoviocytes was significantly lower in OA patients than in controls (Fig. 5).

In addition, although in the KL 2–3 and KL 3–4 subgroups, the miR-146a in PBMCs was significantly higher in OA patients than in controls. There was no significant difference between the two subgroups. Therefore, it cannot be supported that OA KL grade has a significant effect on the expression of miR-146a. Then, although the results of the diagnostic accuracy analysis suggested that plasma miR-146a can help in the diagnosis of OA, there was no significant difference in miR-146a levels between OA patients and controls. Therefore, the diagnostic accuracy of plasma miR-146a needs to be further determined. The rs2910164 and rs57095329 loci of miR-146a also did not show that the mutation would increase the risk of OA.

The key target genes of miR-146a-5p (cumulative weighted context score ≤ − 0.4) included *IRAK1* and *TRAF6,* which are more closely related to the pathogenesis of OA (TargetScan database: http://www.targetscan.org). However, the miR-146a-3p-targeting genes have less evidence to correlate with OA at present. Although the miR-146a SNP-related study did not support that the mutation could increase the OA risk, it suggested that the mutation reduced the expression level of miR-146a and then increased the expression of IRAK1 and TRAF6. Therefore, it confirmed the targeted regulation of miR-146a-5p on IRAK1 and TRAF6 [24].

However, the basic research results of miR-146a and its targeting genes impact OA are still controversial. Studies have suggested that upregulation of miR-146a-5p or inhibition of IRAK1 can alleviate inflammation [42], cartilage degradation [43], and autophagy [44]. Inhibiting TRAF6 expression also alleviated the inflammatory response, reduced the degradation of extracellular matrix [45], and alleviated chondrocyte apoptosis [46]. In addition, in PBMCs, miR-146a-5p also inhibits inflammation through the regulation of IRAK1 [47]. In regulatory T cells, miR-146a-5p exerts immune regulation ability by inhibiting the NF-kb signaling pathway [48]. However, one study showed that miR-146a-5p can promote chondrocyte apoptosis [22].

At present, most viewpoints still believe that miR-146a-5p exerts its inflammatory inhibitory effect by targeting TRAF6 and IRAK1 to inhibit the NF-kb pathway. Therefore, the upregulation of miR-146a mainly plays a protective role in OA development. This might explain the high miR-146a expression level in the early stage of OA and the low level in the late stage [49]. Because the NF-kb pathway also controls miR-146a expression [50], another possible explanation is that miR-146a-5p is a negative feedback molecule of the NF-kb pathway. Therefore, the upregulated expression of miR-146a indicates a higher level of inflammation. Combined with the results of this study, it is suggested that the high expression of miR-146a in PMBCs and cartilage tissue represents a more active NF-kb signaling pathway in patients with OA. A further increase in miR-146a expression may be a means to inhibit the level of inflammation.

### Advantages and limitations

The advantage of this study is that it first evaluated the expression profile of miR-146a in different tissues of OA patients by meta-analysis. This study still has the following limitations. First, this work was performed at the study level instead of at the individual level. Second, data acquisition is mainly obtained from bar charts or scatter charts that might not be accurate enough. Third, there is obvious heterogeneity in the results that might lead to a no robust conclusion. Fourth, the number of studies from some tissue sources is still small, making the results vulnerable to publication bias and tending to be positive.

## Supplementary Information

**Additional file 1**: PRISMA 2020 checklist items for meta-analysis and systematic review.

## Data Availability

All data generated or analyzed during this study are included in this published article.

## Abbreviations

OA: Osteoarthritis
PBMCs: Peripheral blood mononuclear cells
SMD: Standardized mean difference
SNPs: Single nucleotide polymorphisms
RA: Rheumatoid arthritis
qPCR: Quantitative polymerase chain reaction
CI: Confidence interval
SROC: Summary receiver operating characteristic
AUC: Area under the curve
KL: Kellgren–Lawrence
OR: Odds ratio
